# Reversing the pump dependence of a laser at an exceptional point

**DOI:** 10.1038/ncomms5034

**Published:** 2014-06-13

**Authors:** M. Brandstetter, M. Liertzer, C. Deutsch, P. Klang, J. Schöberl, H. E. Türeci, G. Strasser, K. Unterrainer, S. Rotter

**Affiliations:** 1 Photonics Institute, Vienna University of Technology, A-1040 Vienna, Austria; 2 Center for Micro- and Nanostructures, Vienna University of Technology, A-1040 Vienna, Austria; 3 Institute for Theoretical Physics, Vienna University of Technology, A-1040 Vienna, Austria; 4 Institute for Solid-State Electronics, Vienna University of Technology, A-1040 Vienna, Austria; 5 Institute for Analysis and Scientific Computing, Vienna University of Technology, A-1040 Vienna, Austria; 6 Department of Electrical Engineering, Princeton University, Princeton, New Jersey 08544, USA; 7 These authors contributed equally to this work (M.B. (experiment) and M.L. (theory))

## Abstract

When two resonant modes in a system with gain or loss coalesce in both their resonance position and their width, a so-called exceptional point occurs, which acts as a source of non-trivial physics in a diverse range of systems. Lasers provide a natural setting to study such non-Hermitian degeneracies, as they feature resonant modes and a gain material as their basic constituents. Here we show that exceptional points can be conveniently induced in a photonic molecule laser by a suitable variation of the applied pump. Using a pair of coupled microdisk quantum cascade lasers, we demonstrate that in the vicinity of these exceptional points the coupled laser shows a characteristic reversal of its pump dependence, including a strongly decreasing intensity of the emitted laser light for increasing pump power.

## Abstract (web summary)

Exceptional points are singularities that occur when two resonant modes coalesce both in their frequency and width, giving rise to counterintuitive behaviour. Brandstetter *et al*. demonstrate how to induce such an exceptional point in a coupled pair of lasers, causing a reversal in their pump characteristics.

## Graphical abstract

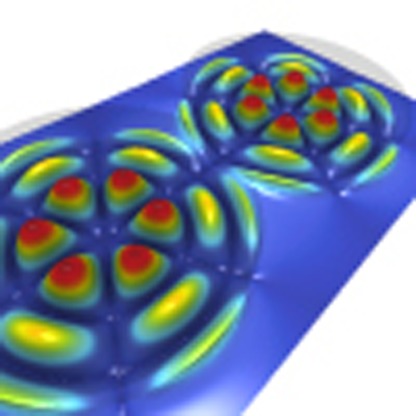

From the standard physics textbook, we know that modes in a closed resonator are conveniently described as the orthogonal eigenstates of a Hermitian operator or matrix, whose real eigenvalues are the corresponding mode frequencies or energies. In the case of resonators, which are open or which feature internal loss or gain, the corresponding matrices are non-Hermitian, featuring complex eigenvalues and non-orthogonal eigenstates. The quasi-bound resonances described by such eigenstates can give rise to so-called exceptional points (EPs), which are non-Hermitian degeneracies at which the real and imaginary parts of two eigenvalues are identical, such that both the position and the width of two resonances are the same. Quite differently from a conventional, Hermitian degeneracy, not only the eigenvalues, but also the eigenvectors coalesce at an EP, leading to a whole host of interesting phenomena^1^,^2^,^3^,^4^, which have recently attracted enormous interest^5^,^6^,^7^,^8^,^9^,^10^,^11^,^12^,^13^,^14^. In particular, experiments on parity-time symmetric systems^15^,^16^, for which the EPs occur on the real frequency axis, have been a driving force behind recent progress^17^,^18^,^19^,^20^,^21^,^22^,^23^,^24^. Already in the first experimental demonstration of this kind^17^, it was shown that a system of two passive waveguides radically changes its transmission, when being steered parametrically through an EP. Whereas earlier work with passive metal cavities^25^ suggests that such a complete reversal of system properties close to an EP is not necessarily restricted to the case of parity-time symmetry, it remains largely unexplored how to observe such a reversal in active optical structures with gain.

The presence of gain changes the rules of the game completely, when it pushes a device across the lasing threshold where the emission of coherent radiation sets in and nonlinear modal interactions build up. An intriguing question to ask is thus in which way the physics of an EP will influence the emission characteristics of a corresponding laser device, such as its lasing threshold, the emission frequency and the corresponding line width or the nonlinear interactions between different laser modes. As all of these concepts do not exist in passive optical structures, research in this direction promises to have many new and fascinating surprises in store that go beyond the physics of EPs below the lasing threshold. First signatures of EPs in lasers have, indeed, already been discussed as, for example, in terms of fast self-pulsations in the laser dynamics near an EP^26^, a diverging laser line width right at the EP^26^,^27^, as well as in relation to characteristic features in a laser’s behaviour below or at threshold^6^,^7^,^14^. Yet, an important milestone still missing in the attempt to connect laser physics with EPs is an experimental demonstration of a similar reversal of system properties as realized for the passive devices discussed above^17^,^25^. That such a counter-intuitive phenomenon can be realized also for lasers is by no means obvious, however. This is mainly because, in contrast to passive systems, lasers above their first oscillation threshold are described by equations, which feature nonlinear terms for the self- and cross-saturation of laser modes^28^. If the contribution of these nonlinear terms dominates over the linear equations that give rise to the EP, also the desired reversal-effect would not be observable. A careful theoretical study is thus required that includes all these contributions quantitatively correctly to clarify under which circumstances a laser’s behaviour can, indeed, be influenced by an EP.

## Results

### Theoretical model

In the corresponding theoretical investigation, which we recently carried out^28^, our starting point was the nonlinear Maxwell-Bloch system of equations that contains the core relations of semi-classical laser theory. Using the newly developed steady-state *ab-initio* laser theory^29^ allowed us to rewrite these equations in their steady-state form, where the lasing modes and frequencies as well as the nonlinear terms, which describe their interactions, appear explicitly. The key insight that we derive from these expressions is that, in a parameter window around the first laser threshold, where the nonlinear terms are still weak, an EP can be induced in the corresponding solutions of these equations by changing the pump applied to the laser. A system that is particularly convenient for realizing such a pump-induced EP turns out to be a device consisting of two coupled lasers. In this specific case, it is sufficient to control the overall pump strength applied to each of the two individual lasers, rather than the spatial pump profile that would have to be carefully modified for setups using just a single laser.

For the purpose of illustrating this idea conceptually, we drastically reduce here the nonlinear lasing equations for this coupled laser (provided in ref. 28) to a simple linear form, where each mode in the laser equations is replaced by a resonant level. Restricting ourselves to only two such levels *A* and *B*, that is, one in each of the two laser resonators, we end up with a 2 × 2 matrix description. The diagonal elements in this matrix describe the levels *A* and *B* with frequencies *a*, *b* and effective gain or loss *α*, *β*. The off-diagonal elements *γ* stand for their coupling,


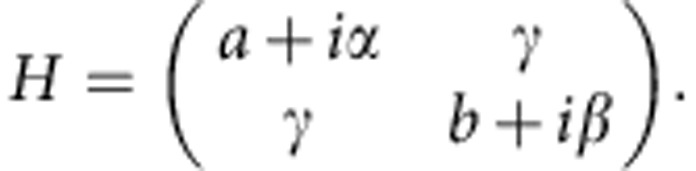


**Figure 1:**
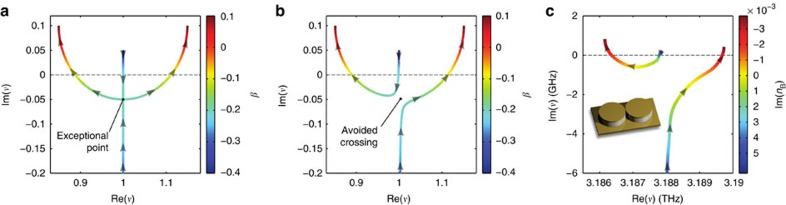
Movement of complex eigenvalues in the vicinity of an EP. The behaviour of a laser with two coupled modes can qualitatively be understood by the eigenvalues of the 2 × 2 matrix *H* given in the text. In **a**, the parametric dependence of these eigenvalues is shown for zero energy splitting between the two modes (*a*=*b*=*1*) and coupling strength *γ*=0.15. When one level is strongly amplified (*α*=0.1) and the other level is swept from being strongly attenuated (*β*=−0.4) to being strongly amplified (*β*→*α*, see arrows), the two eigenvalues first approach each other until they coalesce in an EP. After passing the EP, they are repelled in their real parts and shifted upwards in the complex plane. The real axis (dashed line) represents the lasing threshold and whenever it is crossed by an eigenvalue from above (below) a laser mode turns off (on). Note, that the coalescence of eigenmodes at the EP prohibits an unambiguous labelling of the modes. (**b**) The characteristic reversal of the eigenvalue movement observed in **a** is also seen for a finite frequency splitting (*a=*1.0, *b=*1.002), leading to an avoided eigenvalue crossing in the complex plane. (**c**) Numerical results for the complex resonance frequencies of the experimental device obtained with three-dimensional finite-element calculations. The movement of resonance frequencies under variation of the imaginary part of the index of refraction in disk B shows a similar avoided level crossing as in **b**. (Note that the avoidance of the level crossing as observed here for the coupling of the same modes in two identical disks is due to a complex value of the effective coupling constant *γ*, see refs 48, 49 for details.) An almost degenerate mode pair with a similar eigenvalue movement has been omitted for clarity.

Consider now the situation of two levels with identical frequencies (*a=b*) and real coupling constant γ. Initially, one of the levels (*A*) experiences strong net-gain (*α*>0) and the second one (*B*) features strong net-loss (*β*<0), such that the condition *α*−*β*>>|*γ*| is fulfilled. For this case, the non-Hermitian matrix *H* has one eigenvalue in the upper half of the complex plane and one in the lower half, corresponding to one mode above and the other mode below the lasing threshold (as given here by the real axis). If, in a next step, we continuously add more gain to level *B* until both levels have the same gain (that is, we sweep *β*→*α*), we find the following curious behaviour (see Fig. 1a): first, the two eigenvalues approach each other such that the lasing level is pulled towards the lasing threshold (corresponding to a decrease of light emission). After passing the EP, where the two eigenvalues coalesce, this behaviour changes abruptly and the eigenvalues are repelled in their real parts and then both shifted beyond the lasing threshold (corresponding to an increase of light emission). We may thus conclude that such a device displays exactly the desired reversal of system properties associated with an EP. Note that this behaviour is robust in the sense that it also survives a slight detuning between the two level frequencies (see Fig. 1b) as well as a shift of *γ* into the complex plane (see Fig. 1c). In both cases, the eigenvalues then just pass the vicinity of an EP.

We emphasize at this point that the 2 × 2 matrix description, which we employed above to illustrate our strategy, constitutes a crude approximation of the full problem as realized in the experiment. In particular, all nonlinear effects related to mode competition, gain pulling, as well as any temporal instabilities and the influence of other than the two considered modes are entirely neglected in this toy model. Also the frequency- and pump-dependence of the coupling between the two modes as well as all effects specific to the quantum cascade lasers (QCLs), which we work with in the experiment, are completely ignored, such that this model only has qualitative rather than quantitative predictive power. Still, as we will show below, our simple 2 × 2 matrix model can capture the essential physics of an EP quite well. To model the experiment quantitatively correctly, however, an appropriate treatment of the three-dimensional nature of the resonators and of their coupling is required (as provided below). An accurate description of the laser’s above-threshold behaviour necessitates, in addition, a fully nonlinear calculation (as provided in ref. 28).

### Coupled microcavity QCLs

A natural experimental arena in which the above lasing effect could be realized is given by coupled ridge or microcavity lasers^30^,^31^ in each of which one level can be brought to lase. A direct implementation of such a photonic molecule laser, however, meets a number of challenges: first of all, the coupled laser needs to operate in the single-mode regime, as the onset of additional modes would give rise to nonlinear mode-competition effects, which may overshadow the effect of an EP. Owing to this requirement, the gain coefficient *α*>0 from above is restricted to rather small values for which only one mode is lasing in the first disk. Second, to realize the initial gain/loss-configuration *α*−*β*>>|*γ*|, from which we start the pump sweep (*β*→*α*), the loss coefficient *β*<0 must be very negative (for *α* being small), corresponding to strong absorption in the second disk. Third, the laser geometries as well as the coupling gap between them need to be engineered on the scale of the lasing wavelength such as to obtain sufficiently similar resonator frequencies as well as the right coupling strength.

**Figure 2:**
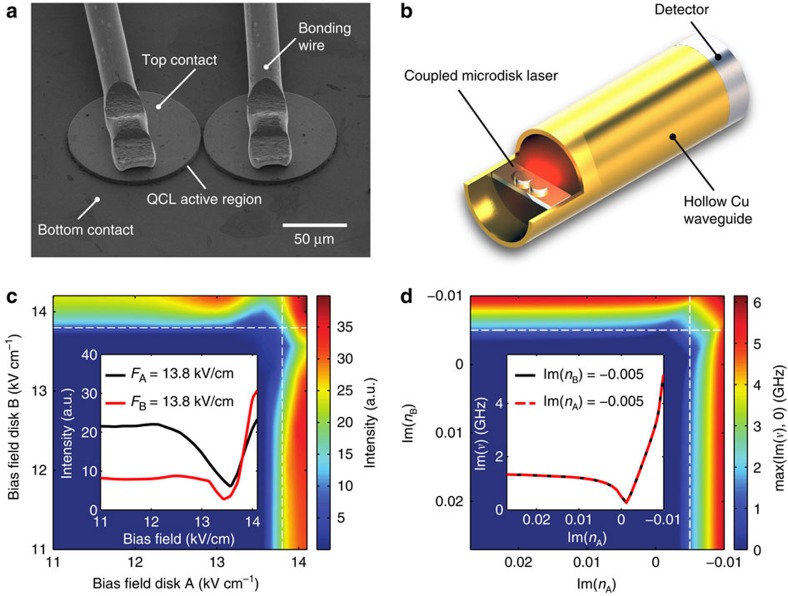
Photonic molecule laser and its pump dependence. (**a**) Image of the studied photonic molecule quantum cascade laser taken with a scanning electron microscope (disk radius *r=*47 μm, height *h=*3.5 μm, inter-cavity distance *d=2 *μm, Ti absorption layer thickness *d*_abs_=90 nm). (**b**) Configuration for spatially integrated measurements. A high collection efficiency is obtained by guiding the emitted light to the detector using a hollow metallic waveguide. (**c**) Measured intensity output from the photonic molecule laser in **a** (integrated over all frequencies and emission directions) as a function of the electric field strength applied to the two individual disks (in the dark blue region the laser is off). The upper right corner contains the non-monotonic pump dependence expected for an EP. When the field strength in one of the disks is fixed and the other disk is steered through the EP’s vicinity (see white dashed lines), this results in a characteristic reversal of the laser’s pump dependence (see inset for the corresponding intensity curves). (**d**) Numerical results from the three-dimensional simulations: The maximum of the positive imaginary parts of the complex resonance frequencies is shown as a function of the amplification in each disk, as given by the corresponding imaginary parts of the refractive indices (only the *n=*3 modes are considered which are lasing in the experiment). Note the excellent agreement that we find between these calculations and the experimental data in **c**.

Systems that we found to fulfil all of the above stringent criteria are photonic molecule QCLs operating in the THz regime^30^,^32^,^33^. Here, the gain is produced by transitions between quantized energy levels of semiconductor quantum wells, allowing us to adjust the emission wavelength by the quantum well widths. In our experiment, the emission wavelength (~100 μm) is comparable to the size of the actual coupled microdisk lasers, such that these devices feature both a stable regime of single mode operation as well as a much higher fault tolerance with respect to geometric imperfections as compared with corresponding lasers emitting in the visible spectrum of light. Furthermore, QCLs are electrically pumped devices; they provide a symmetric Lorentzian gain profile^34^ (in contrast to bandgap semiconductor lasers) and do not suffer from surface recombination. Specifically, we fabricated pairs of disk-shaped lasers, which we placed in close vicinity to each other in order to achieve sufficiently strong mode coupling. The active region of the laser is sandwiched, on top and at the bottom, by two metal layers, which act both as a waveguide and as a contact for electrically pumping the device. Owing to their finite conductivity, these metal layers provide much of the required loss already quite naturally. As, however, even higher loss values are necessary to observe an EP, we reduced the thickness of the active region and added an additional absorption layer (see Methods section). Figure 2a shows an image of a fabricated device.

### Measurements

In a first step, we performed measurements on the total emitted light intensity of this coupled laser device. Using the setup depicted in Fig. 2b, we collected the emitted laser light through an oversized hollow metallic waveguide, which guides the light to the detector resulting in almost 50% collection efficiency. Owing to the symmetry of the setup, the emitted radiation, which leaves the waveguide in the other direction, has the same intensity and is thus not detected. An advantage of this spatially and spectrally integrated measurement is that the results are insensitive to specific details of the far-field emission pattern and of the emission frequency of the laser. The pump dependence of this photonic molecule laser that we measure in this way is shown in Fig. 2c, where the emitted laser light intensity (see false colour plot) is displayed as a function of the bias fields *F*_A_ and *F*_B_ applied to disks A and B, respectively. In this plot, the specific pump trajectory, which induces an EP along the discussion above, is realized both as a vertical and as a horizontal line (see insets). In both of these configurations, the starting point is such that the applied bias is above threshold in one disk and below in the other disk. When gradually adjusting the lower field value to the higher value, the laser, indeed, shows the characteristic behaviour, which we predicted: after a regime where an increase of the bias field does not influence the output intensity at all, we first observe a strong reduction in the emitted light intensity, followed by an increase beyond the initial value. The observation of a reversal in the laser’s pump dependence constitutes a clear signature of the presence of an EP near those parameter values where the reversal occurs. To rule out that the observed behaviour is caused by some other mechanism, we carried out a number of additional checks.

**Figure 3:**
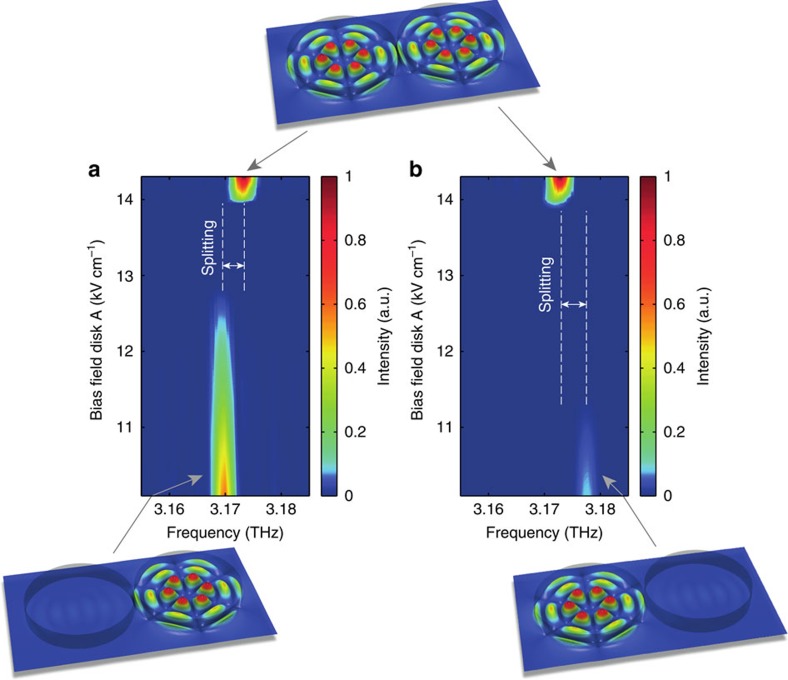
Emission spectrum and lasing modes. (**a**) Experimental emission spectrum recorded with the device in Fig. 2a, as a function of the pump strength in disk A with disk B at a constant pump value slightly above the lasing threshold. The colour gradient is proportional to the measured intensity and shows that along this pump sweep a single lasing line first turns off and then re-emerges with a shifted frequency (see frequency splitting indicated by the double-arrow). Note that the measured line width is limited here by the resolution of the spectrometer. (**b**) When varying instead the pump strength in disk B with disk A slightly above threshold, the equivalent behaviour is observed, however, with a frequency shift of opposite sign. The insets depict the electric field profiles (*|E(x)|*) of the involved modes as calculated at the bottom and top end of the pump sweeps shown in **a**,**b**.

As a first test, we performed extensive numerical simulations of the studied setup to verify the presence of the EP in the complex eigenvalue surfaces explicitly (see Methods section). As the coupling between two disks is spuriously overestimated in two-dimensional scalar calculations, we performed three-dimensional vectorial simulations of the photonic molecule device. To emulate the effect of the varying pump strength in the experiment, we solved the Helmholtz equation for the resonances of the photonic molecule under variation of the imaginary part of the index of refraction *n*_A_ and *n*_B_ of the two respective microdisks. The first insight derived from these calculations is that the modes which start lasing first in the experiment are whispering gallery modes with radial quantization number *n*=3 (see insets in Fig. 3 and Supplementary Figs 1 and 2). For these modes, a variation of the imaginary part of the refractive index in one disk, for example, Im(*n*_B_), yields the expected avoided level crossing (see Fig. 1c) as previously obtained with the 2 × 2 matrix model (see Fig. 1b). When varying the imaginary parts of both refractive indices, Im(*n*_A_) and Im(*n*_B_), we obtain the same characteristic dependence on these parameters (see Fig. 2d) as observed in the experiment when varying the applied field strengths *F*_A_ and *F*_B_ (see Fig. 2c).

As a final test, we also successfully verified that our experiment reproduces the real frequency shift which modes experience when passing the vicinity of the EP. Following the graphical illustration in Fig. 1, this shift should be observable as a frequency splitting between the lasing modes before and after they pass the EP. To check this behaviour explicitly, we recorded spatially integrated but spectrally resolved emission data, where the pump bias applied on one disk was fixed at a value slightly above threshold and the bias applied on the other disk was swept through. The recorded data shown in Fig. 3a clearly demonstrate that the laser line has a well-resolved splitting between the lasing frequency before and after the EP-induced laser shutdown. The insets of this figure illustrate the spatial mode patterns that are realized at the respective pump conditions. To verify the left–right symmetry of our setup, we also performed the same measurement with the role of the two disks interchanged (see Fig. 3b). Also in this case, we find the desired frequency splitting as well as the laser shutdown at the EP. As already suggested by the plots in Fig. 1, we observe in Fig. 3a,b that the mode frequencies can be shifted to higher as well as to lower values when passing the EP. The data contained in Fig. 3 also clearly demonstrate that the entire recorded pump sweep across the EP involves just a single lasing line, which is switched off and then on again. Nonlinear mode competition effects are therefore ruled out as an explanation for the shutdown of the laser. Such nonlinear effects do, however, become important when going farther above threshold, as at the end of the pump sweep, where both disks are pumped equally and strongly. In this case, the linear theory in Fig. 1c predicts two modes above the laser threshold, whereas in the experiment only a single mode is observed. Our numerical results suggest that the suppression of the second mode is due to modal cross-saturation (spatial hole burning).

## Discussion

The above results allow us to unambiguously identify the influence of an EP on the operation characteristics of a photonic molecule laser. Coupling two electrically pumped QCLs with each other and tuning the voltages applied on each of the two constituent laser disks lets us conveniently steer this coupled device through the vicinity of an EP. We observe very counter-intuitive lasing effects, such as a previously predicted characteristic reversal of the laser’s pump dependence, which results from the movement of complex eigenvalues close to the EP. This observation establishes photonic molecule lasers as promising tools for exploring many further fascinating aspects of EPs, like a strong line-width enhancement^5^,^26^,^27^ and the coherent perfect absorption of light^6^,^7^ in their vicinity as well as non-trivial mode-switching^8^ and the accumulation of a geometric phase^9^ when encircling an EP parametrically. A recent experimental study also shows how such concepts can be transferred from optics to electronics, where the photonic molecule laser we use is replaced by two coupled electrical circuits^35^.

We expect that our work will also be of practical relevance. This is because the advanced QCLs, which we study, are in use already in several applications^36^,^37^,^38^,^39^, where the feature that they are pumped electrically offers the prospect of realizing lab-on-a-chip instruments^40^. In view of the ever-increasing packaging density of optical devices on such chips, our new insights on the coupling between two or several lasers that are integrated on them will be essential. The strong dependence of the physics of EPs on the coupling strength and on optical losses is also very attractive for highly accurate optical-sensing applications^41^. Our results thus not only exemplify interesting EP physics with lasers, but also open up new opportunities for the realization of electrically controllable photonic devices with unconventional optical properties.

## Methods

### Active region and fabrication

A QCL-active region consists of multiple coupled quantum wells, forming quantized energy levels, which, in turn, act as laser states. The emission wavelength can thus be adjusted by designing the well and barrier widths and is therefore material independent. As QCLs are unipolar devices (only electrons are involved in the carrier transport), surface recombination effects do not affect the laser operation, which enables us to fabricate resonator structures with arbitrary geometry^42^ and sub-wavelength dimensions^43^.

The QCL-active region used here is realized in the GaAs/Al_0.15_Ga_0.85_As material system, grown with molecular beam epitaxy. It is based on a 3-well LO phonon depletion design^44^ with a designed emission frequency of 3.2 THz. The layer sequence starting from the injector barrier in nanometers is **4.8**/9.6/**2**/7.4/**4.2**/16.1, the 16.1-nm well is homogeneously n-doped with a density of 7.5 × 10^15^ cm^−3^. The QCL module is repeated 271 times giving a total thickness of 13 μm. In order to improve the electrical contact, highly doped GaAs layers were grown on top and at the bottom of the active region with a thickness of 50 and 100 nm, respectively. For the fabrication of the devices in the double-metal waveguide^45^, we covered the active region and a carrier substrate with a 1-μm-thick gold layer by magnetron sputtering. In the subsequent Au-Au thermo-compression bonding step, we attach the active region to the carrier substrate. The GaAs substrate, on which the QCL was grown onto, is removed wet chemically.

We reduced the thickness of the structure to increase the waveguide losses using a dry-chemical process by reactive ion etching, providing a smooth surface and homogeneous etch rates across the sample. We define the gold top contact/waveguide layer and an optional Ti absorption layer by a photo-lithography/lift-off process, acting as a self-aligned etch mask for the subsequent reactive ion etching process, defining the resonator. The devices are then mounted to a copper heat sink with indium and contacted using a wire-bonding technique.

### Measurement setup

All measurements are performed in continuous wave mode in order to achieve stable operation conditions. For the integral measurements of the bias-dependent intensity plot in Fig. 2c, the samples are mounted on a probe, which is put directly in a liquid helium Dewar to efficiently cool the devices and to obtain a reproducible and stable temperature, which is important for continuous wave operation. The emitted intensity is measured using a Ga-doped Ge detector. The spectral measurements are performed using a Bruker Vertex 80 FTIR spectrometer with a resolution of 2.25 GHz. The emitted light is recorded with an attached pyroelectric deuterated triglycine sulfate detector. The sample is mounted in a liquid helium cooled flow cryostat, attached to the spectrometer.

### Numerical mode calculation

The numerical results for the resonant modes and the associated complex resonance eigenvalues are obtained by solving the fully vectorial three-dimensional Helmholtz equation,


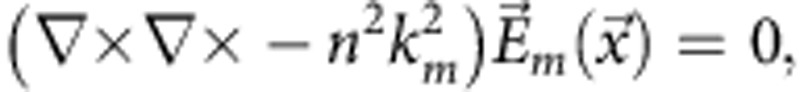


with open (outgoing) boundary conditions. The real part of the index of refraction *n=*3.61 has been determined by comparing theoretical calculations and experimental measurements of devices featuring multiple laser modes (see Supplementary Figs 1 and 2). The imaginary part of the index of refraction is varied as shown in Figs 1 and 2. The metal waveguide layers were approximated as perfect conductors and the outgoing boundary conditions are imposed using a perfectly matched layer. The dimensions of the simulated photonic molecule are the same as in the experiment. The calculations were carried out using the open source library *ngsolve*^46^, which was developed by one of the authors (J.S.) based on a high-order finite element discretization technique.

### Waveguide loss calculation

For the calculation of the waveguide loss in a double-metal structure, we make use of a one-dimensional waveguide solver based on a standard transfer-matrix formalism. As the QCL structure consists of 25% Al_0.15_Ga_0.85_As, we model the active region using the mean value of the complex refractive indices, with a mean doping density of 5.3 × 10^15^ cm^−3^. The 50-nm thick GaAs contact layer on the bottom of the active region is assumed to have a doping density of 5 × 10^18^ cm^−3^. The simulation model consists of the active region stacked between two gold contact layers and a Ti absorption layer between the active region and the top contact layer. The material parameters of Ti are described using a Drude-model with a plasma frequency *ω*_p_=2*π*·6.09 × 10^14^ s^−1^ and a damping frequency of *ω*_t_=2*π*·1.14 × 10^13^ s^−1^, the parameters for gold are taken as^47^ *ω*_p_=2*π*·2.18 × 10^15^ s^−1^ and *ω*_t_=2*π*·6.44 × 10^12^ s^−1^. Our model shows that the waveguide losses increase both with increasing absorption layer thickness and with decreasing waveguide height, which is due to a larger influence of the metal layers with finite conductivity. A certain minimum thickness of the active region is necessary, however, as the coupling strength of the disks is reduced for thinner waveguides (due to the strong diffraction of the emitted light at small apertures). The absorption layer is thus needed to achieve both the right amount of losses and a strong enough coupling simultaneously. Based on these considerations, the device we designed for the experiment had a waveguide height *h*=3.5 μm and an absorption layer thickness of *d*_abs_=90 nm for which our model yields waveguide losses of 47 cm^−1^.

## Additional information

**How to cite this article:** Brandstetter, M. *et al*. Reversing the pump dependence of a laser at an exceptional point. *Nat. Commun.* 5:4034 doi: 10.1038/ncomms5034 (2014).

## Author contributions

M.B. and C.D. performed the experiments, M.L. carried out the numerical calculations with the help of J.S., who also wrote the numerical code (ngsolve). P.K. and G.S. grew the quantum cascade hetero-structure. S.R., K.U. and H.E.T. initiated and supervised the project at all stages. M.B., M.L. and S.R. wrote the first manuscript draft. All authors discussed the results and commented on the manuscript.

## Supplementary Material

Supplementary Information Supplementary Figures 1-2
