# Supplementary material for: Reversing the pump dependence of a laser at an exceptional point
Source: Nat Commun. 2014 Jun 13;5:4034. doi: 10.1038/ncomms5034 (PMC4082637; doi:10.1038/ncomms5034)
Supplement: Supplementary Information — Supplementary Figures 1-2 [file ncomms5034-s1.pdf]

Supplementary Figure 1

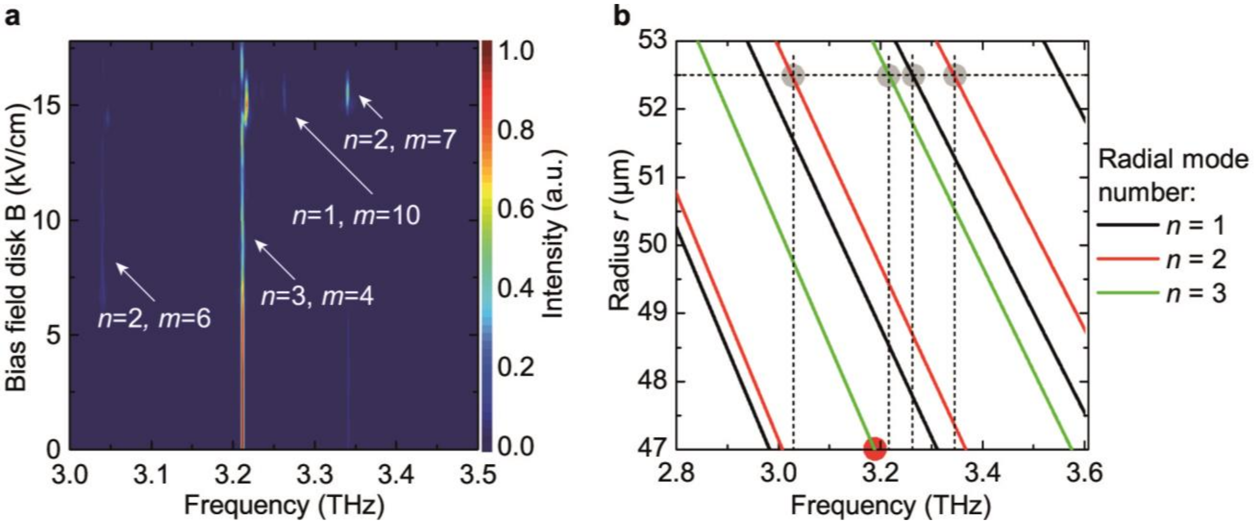

Identification of the measured modes. a, Measured spectrum of a device showing multi-mode emission due to processing imperfections (radius r=52.5 µm, waveguide height h=4.5 µm, inter-cavity distance d=2 µm, bias field at disk A F_A=14.9 kV/cm). The comparison to a 3D simulation using a finite element solver, shown in **b**, allows us to identify the measured modes, which are labelled by the radial and azimuthal mode numbers n and m. We assume a refractive index of the active region of n_r = 3.61. The black, red and green lines indicate modes with n = 1, 2 and 3 radial maxima, respectively. The grey circles highlight the positions of the experimentally obtained modes, which perfectly fit to the simulation. The red circle indicates the mode occurring in the device presented in the main text, showing 3 radial maxima.

Supplementary Figure 2

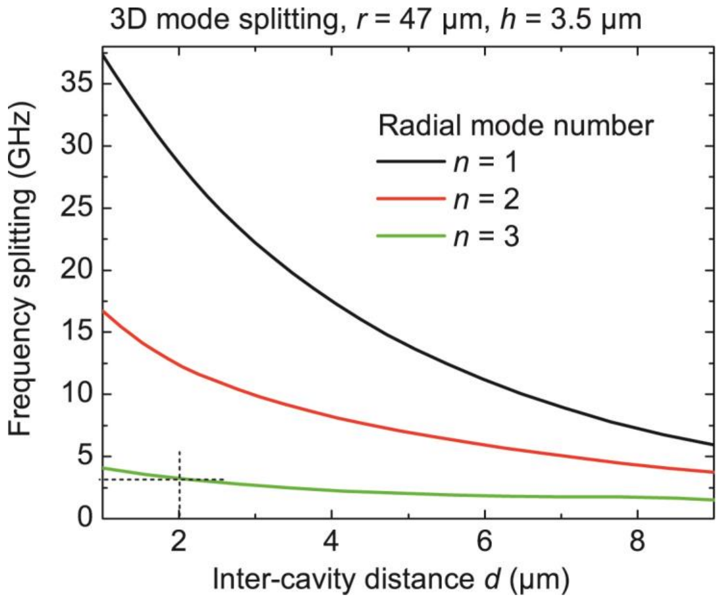

Calculated mode splitting of coupled disk modes. The real mode splitting in the coupled cavity system is shown for modes with radial mode number n=1, 2 and 3 as a function of the inter-cavity distance d. In the corresponding 3D FEM calculations the device dimensions are chosen to be the same as used in the experiment (radius r=47 µm, waveguide height h=3.5 µm). The dashed lines indicate the values for d=2 µm, resulting in a mode splitting of 3.2 GHz. The experimental value of 3.9 GHz for the same value of d=2 µm is in good agreement with the simulation and thus confirms our mode labeling. Note that a lower radial mode number would lead to a significantly larger splitting.
